# First report of QNRA isolated from extended spectrum B-lactamase producing hospital-acquired *Klebsiella pheumoniae* in Kuwait

**DOI:** 10.1186/1753-6561-5-S6-P139

**Published:** 2011-06-29

**Authors:** L Vali, AA Dashti, MM Jadaon, S El-Shazly, BT Jose

**Affiliations:** 1 Kuwait University, Kuwait, Kuwait

## Introduction / objectives

Extended Spectrum beta-lactamase (ESBL)-mediated resistant *Klebsiella pneumoniae* are important opportunistic pathogens. In this study we investigated the prevalence of plasmid-mediated fluoroquinolone resistance in ESBL-producing *K. pneumoniae* in nosocomial infections in Kuwait.

## Methods

From a total of 72 non-duplicate quinolone and cephalosporin resistant *Enterobacteriacae* obtained from October-December 2010 from Ahmadi hospital in Kuwait, 16 were *K. pneumoniae*. Antimicrobial susceptibility was determined by Vitek, Microscan, double disc diffusion, agar dilution and E-test against a panel of 26 antimicrobial agents. The presence of *bla*SHV, *bla*TEM, *bla*CTX-M, *gyr*A, *par*C, *qnr*A, *qnr*B, *qnr*S and class 1 integrons were determined by PCR and sequencing. Pulsed-field gel electrophoresis (PFGE) was used for typing the strains and the results were analysed according to Tenover criteria.

## Results

All 16 isolates were resistant to all antibiotics tested including ciproflaxoacin (MIC>4), tazobactam (MIC>16), cefotaxime (MIC>16) and ceftazidime (MIC>16); except for carbapenems, amikacin, and tigecycline. *bla*TEM, *bla*SHV& &*bla*CTX-M-15 were present in 81.25% (13), 81.25% (13) and 68.75% (11) respectively. Nine (56.25%) isolates contained all three *bla* genes of which one harboured *qnr*A (A2 allele) and a class 1 integron. No mutations were found in *gyr*A and *par*C. PFGE revealed that *K. pneumoniae* isolates harbouring ESBL genes consisted of two distinct clones.

## Conclusion

Contrary to a previous study, we hereby report the emergence of plasmid-mediated *qnr*A gene among ESBL producing nosocomial *K. pneumoniae* for the first time in Kuwait. Identification of these strains are crucial for administering the correct antibiotic and preventing their spread among hospitalised patients.

## Disclosure of interest

None declared.

